# Informing a European guidance framework on electronic informed consent in clinical research: a qualitative study

**DOI:** 10.1186/s12913-023-09173-5

**Published:** 2023-02-21

**Authors:** Evelien De Sutter, Pascal Borry, Isabelle Huys, Liese Barbier

**Affiliations:** 1 Clinical Pharmacology and Pharmacotherapy, Department of Pharmaceutical and Pharmacological Sciences, KU Leuven, Leuven, Belgium; 2 Centre for Biomedical Ethics and Law, Department of Public Health and Primary Care, KU Leuven, Leuven, Belgium

**Keywords:** Clinical research, Trial, Digital technology, Stakeholders, Guideline, Implementation, Informed consent, Regulatory guidance, Europe

## Abstract

**Background:**

Electronic informed consent (eIC) may offer various advantages compared to paper-based informed consent. However, the regulatory and legal landscape related to eIC provides a diffuse image. By drawing from the perspectives of key stakeholders in the field, this study aims to inform the creation of a European guidance framework on eIC in clinical research.

**Methods:**

Focus group discussions and semi-structured interviews were conducted with 20 participants from six stakeholder groups. The stakeholder groups included representatives of ethics committees, data infrastructure organizations, patient organizations, and the pharmaceutical industry as well as investigators and regulators. All were involved in or knowledgeable about clinical research and were active in one of the European Union Member States or at a pan-European or global level. The framework method was used for data analysis.

**Results:**

Stakeholders underwrote the need for a multi-stakeholder guidance framework addressing practical elements related to eIC. According to the stakeholders, a European guidance framework should describe consistent requirements and procedures for implementing eIC on a pan-European level. Generally, stakeholders agreed with the definitions of eIC issued by the European Medicines Agency and the US Food and Drug Administration. Nevertheless, it was raised that, in a European guidance framework, it should be emphasized that eIC aims to support rather than replace the personal interaction between research participants and the research team. In addition, it was believed that a European guidance framework should include details on the legality of eIC across European Union Member States and the responsibilities of an ethics committee in the eIC assessment process. Although stakeholders supported the idea to include detailed information on the type of eIC-related materials to be submitted to an ethics committee, opinions varied on this regard.

**Conclusion:**

The creation of a European guidance framework is a much needed factor to advance eIC implementation in clinical research. By collecting the views of multiple stakeholder groups, this study advances recommendations that may facilitate the development of such a framework. Particular consideration should go to harmonizing requirements and providing practical details related to eIC implementation on a European Union-wide level.

**Supplementary Information:**

The online version contains supplementary material available at 10.1186/s12913-023-09173-5.

## Background

Clinical trials, an essential part of the drug lifecycle, intend to add knowledge to health-related biomedical or behavioral outcomes of a medical intervention [1, 2]. While generally essential in the drug lifecycle, shortcomings of traditional clinical trials have been described, for example, related to the required number of participants to achieve the study objectives or the risk of failure, which may hamper the development of medical interventions [3, 4]. As a result, focus has been put on novel approaches to design clinical trials, such as making use of master protocols [4, 5]. In addition, technological advancements are catalyzing the conduct of clinical trials from a traditional clinical site to a remote setting [6, 7].

Electronic informed consent (eIC), one of these technological advancements, refers to providing trial-related information to research participants and obtaining their consent electronically [8]. Benefits of eIC, complementing the verbal elucidation provided by the research team, have been argued to be manifold [9–11]. First, eIC may empower participants to make an informed decision on taking part in a trial through a personalized approach. For instance, trial-related information can be conveyed through multimedia elements, tailored to the research participants’ needs [10, 11]. Next to this personalized approach, eIC may facilitate ongoing interaction between the participants and the research team, for example, for providing the trial results [9]. In addition, eIC may support quality assurance by automatically generating date/time stamps and ensuring correct versioning [9, 12].

Whilst eIC may offer various advantages compared to paper-based informed consent forms, there are questions about practical elements related to the implementation of eIC in clinical research, such as the legality of eIC throughout European Union (EU) Member States and its impact on the ethical review process [10]. Although the Clinical Trials Regulation (CTR) facilitates harmonization of the conduct of clinical trials throughout EU Member States, it does not specifically address eIC [13, 14]. The Regulation on electronic identification and trust services for electronic transactions in the internal market lays down the requirements for electronic signatures. According to this Regulation, a qualified electronic signature is the legal equivalent of a handwritten signature [15]. However, this Regulation does not specifically target the use of electronic signatures in clinical research and thus, the legality of obtaining the participants’ consent via electronic means may vary across EU Member States [13].

Over the past years, guidance documents addressing the implementation of eIC in clinical research have been issued by regulatory bodies. In 2016, the US Food and Drug Administration (FDA) published a guideline setting out the requirements for seeking and documenting eIC in clinical investigations [16]. In the light of the COVID-19 pandemic, the European Medicines Agency (EMA) issued guidance to support the continuation of clinical trials. This guidance states that “*any validated and secure electronic system already used in the trial in the particular member state for obtaining informed consent can be used as per usual practice and if in compliance with national legislation*” [17]. In addition, a draft guideline on computerized systems and electronic data in clinical trials was released by the EMA in 2021, aiming to support stakeholders in complying with the relevant regulatory and legal requirements. This guideline, which was opened for public consultation, further specifies the use of eIC in clinical trials; for example, related to conveying trial-related information to research participants and access to the informed consent documentation prior and after obtaining their consent [8].

The latter guideline may be a stepping stone towards wider adoption of eIC across EU Member States [8]. However, a number of important issues remain. It remains unclear what kind of European guidance framework would ideally be needed to facilitate harmonization and how national guidance should relate to such a European framework. In addition, practical elements that could be addressed in a European guidance framework demand further attention, for example, related to the legality of eIC and the ethical review process. Given the multi-stakeholder nature of (electronic) informed consent, input on these issues should be gathered from all actors involved (i.e., investigators, regulators, and representatives of ethics committees (ECs), data infrastructure organizations, patient organizations, and the pharmaceutical industry). The insights provided by these stakeholder groups may inform the creation of a European guidance framework related to the implementation of eIC in clinical research.

## Methods

This qualitative study adheres to the Consolidated Criteria for Reporting Qualitative Research (COREQ) recommendations (Additional file 1) [18].

### Design

A qualitative study was designed, involving focus group discussions and semi-structured interviews, to investigate the perspectives of various stakeholder groups active in EU Member States or at a pan-European or global level. A focus group discussion was chosen to facilitate interaction and information exchange between stakeholders [19, 20]. In addition, semi-structured interviews were conducted to tap further into the expert knowledge of stakeholders, who could express themselves individually without constriction. The topic guide used during the focus group discussions and semi-structured interviews was informed by previous research and the research aims. Based on a systematic literature review and semi-structured interviews with various stakeholder groups involved in clinical research, uncertainties related to eIC were identified that need further discussion [9, 10]. These uncertainties were related to (i) the legality of eIC, (ii) the eIC materials that need to be submitted to the EC, (iii) the responsibilities ECs have when assessing eIC, and (iv) the conduct of the eIC process (e.g., whether it can take place via telephone calls). Hereafter, it was investigated whether and how these uncertainties were addressed in the guidance documents of the EMA (*draft ‘Guideline on computerised systems and electronic data in clinical trials*’) and the FDA (‘*Use of electronic informed consent in clinical investigations – Questions and answers*’) [8, 16]. This investigation further informed the design of the topic guide. The topic guide followed a semi-structured format and consisted of questions around (i): the definition of eIC, (ii) the type of European guidance framework required, and (iii) the abovementioned uncertainties (Additional file 2).

### Participants

A purposeful sampling approach was used to select the following key stakeholders involved in clinical research: investigators, regulators, and representatives of ECs, data infrastructure organizations, patient organizations, and the pharmaceutical industry. A purposive sample was gathered by searching stakeholder websites and via the research group’s network. In addition, we asked the enrolled stakeholders to disseminate our study invitation to other participants of interest and refer us to eligible contacts (i.e., snowballing). Eligible participants needed to be (i) active in an EU Member State or at a pan-European or global level, (ii) involved in or knowledgeable about clinical research, and (iii) fluent in English. An invitation containing the informed consent for study participation and data processing was mailed to suitable participants. Recruitment continued until no new themes emerged, indicating that data saturation was achieved [21].

### Data collection

Focus group discussions and semi-structured interviews were conducted via Microsoft Teams between November 2021 and August 2022. In addition, focus group discussions and interviews were audio-recorded, conducted in English or Dutch, and were 1–2 h and 30–50 min long, respectively. Prior to participation in the focus group discussion or semi-structured interview, participants were provided with the topic guide and, for the sake of completeness, with the guidance documents issued by the EMA and the FDA [8, 16]. At the beginning of each interaction, the moderator (JD or EDS) introduced him- or herself and explained the purpose and procedures. Next to a moderator, one or two observers (LB and JD or EDS) were present in the focus group discussions and were responsible for time keeping and for taking notes. A PowerPoint presentation was used to guide participants through the focus group discussion or semi-structured interview.

### Data analysis

The audio-recordings were transcribed verbatim by the researchers (EDS, JD, and LB) and by a third-party. The data obtained were analyzed deductively (i.e., based upon the research aims and the topic guide) and inductively (i.e., based upon observed patterns) utilizing thematic analysis, through a process of data familiarization and generating codes using NVivo software [22]. JD coded the first transcript and discussed regularly with the other researchers (EDS and LB) to refine and agree on the defined codes. Hereafter, a working analytical framework, also known as a coding tree, was created which was then applied to the other transcripts by one researcher (EDS) (Additional file 3). Finally, the coded data were charted into a matrix using Microsoft Excel and were then interpreted.

## Results

### Participant characteristics

In total, two focus group discussions were conducted: (i) a multi-stakeholder focus group discussion (n = 5) involving one EC representative, one investigator (who was also an EC representative), one representative of a data infrastructure organization, and two patient organization representatives, and (ii) a focus group discussion with EC representatives only (n = 6). In addition, eight semi-structured interviews were held with regulators (n = 5) and pharmaceutical industry representatives (n = 4, of whom two interviewees were present at a single interview). Participants across the stakeholder groups were working in Austria, Belgium, Denmark, Finland, Italy, Lithuania, the Netherlands; or were active at a pan-European or global level (Table 1).

**Table 1:** Characteristics of participants in the focus group discussions and semi-structured interviews (n participants = 20, n interviews = 19)

	Participant details
Focus group discussion 1 (n participants = 5)	One representative of a data infrastructure organization active on a global level, one EC representative active in Belgium, one investigator (who was also an EC representative) involved in a pan-European organization, and two patient organization representatives active at a pan-European level.
Focus group discussion 2 (n participants = 6)	EC representatives were active in Belgium (n = 3), Denmark (n = 1), Lithuania (n = 1), and the Netherlands (n = 1). Two representatives were also active at a pan-European level or in a national umbrella organization.
Semi-structured interviews (n participants = 9, n interviews = 8)	Regulators were active in Austria (n = 1), Finland (n = 1), Italy (n = 1), Lithuania (n = 1), and the Netherlands (n = 1). Two regulators were also involved in a pan-European organization. Representatives of the pharmaceutical industry were involved in clinical research on a Belgian (n = 1), European (n = 1), and global (n = 2) level.

### High-level considerations on the development of a European guidance framework

#### The need and value of a European guidance framework

Almost all participants across stakeholder groups perceived the EMA draft ‘Guideline on computerised systems and electronic data in clinical trials’ as a core document in the field. Several pharmaceutical industry representatives and the data infrastructure organization representative asserted that this guideline will help to alleviate ambiguity and uncertainty about eIC implementation. Some EC representatives and regulators raised that the EMA guideline could function as a reference document during the assessment of eIC.

“*The assessment process [by regulatory bodies] will be facilitated if the study protocol would state that certain guidelines were met. In that case, we have more certainty about the eIC system used in a clinical trial.*” (13, regulator).

However, multiple EC representatives argued that the EMA guideline is a rich source of information for sponsors rather than for ECs. They raised the point that the guideline is a relatively technical document that will have a limited place in their evaluation process.

“*We were very optimistic when we first heard about a guideline issued by the EMA. We had high expectations to find answers to very practical issues raised in daily practice, which was unfortunately not the case.*” (9, EC representative).

Similarly, some regulators and pharmaceutical industry representatives mentioned that it concerns a comprehensive guide on computerized systems and electronic data in clinical trials and that, for eIC implementation, additional, more specific issues have to be addressed. To this end, it was raised that a European guidance framework is needed, beyond an EMA guideline, that should include more practical details related to eIC (e.g., related to the ethical review process of eIC). In addition, the majority of stakeholders agreed that a European guidance framework on eIC should have a high value to facilitate harmonization of eIC implementation in practice and thus, support an efficient conduct of multi-country clinical trials.

“*We have eIC deployed in many clinical trials of which multiple involved more than 15 countries. The differences at national level are enormous and the burden to starting up studies and getting the EC approvals is absolutely horrendous.*” (1, data infrastructure representative).

The investigator and a patient organization representative argued that a European guidance framework should be established by means of a regulation. On the other hand, many other participants across stakeholder groups reported that it would be more feasible to create and implement harmonized guidelines, for example, via good research practices or recommendations, that may ultimately result in legislation.

“*We are in such an early stage with eIC. I think it would be difficult to have a framework that has a legislative and regulatory value. I believe that providing recommendations is the best we can do until the use of eIC is more mainstream.*” (18, pharmaceutical industry representative).

One regulator referred to the recommendations related to contraception and pregnancy testing in clinical trials, issued by the Clinical Trials Facilitation and Coordination Group (CTFG), and raised that a similar approach could be undertaken for eIC. In addition, this particular regulator as well as a pharmaceutical industry representative considered recommendations easier to amend in comparison to a legally binding act, which was deemed useful in the view of evolving technology.

#### Towards a multi-stakeholder guidance framework

All stakeholders agreed that multi-stakeholder partnerships are needed, involving patient organizations, regulatory bodies, policy makers, health care professionals, sponsors, ECs, data protection authorities, and clinical research organizations to design a European guidance framework on eIC.

“*We really think that data protection authorities should be involved in the design of a European guidance framework in order to incorporate the relevant data protection requirements.*” (20, pharmaceutical industry representative).

Many participants across stakeholder groups referred to the Clinical Trials Expert Group (CTEG) of the European Commission or the EMA to take a leading role in designing such a framework. A particular EC representative emphasized that, when multiple stakeholder groups are involved, it is of utmost importance that they share similar objectives.

“*Before starting this joint effort, we have to be sure that all stakeholders share the same objectives behind developing a framework and implementing eIC.*” (7, EC representative).

#### A European versus a national guidance framework

When asked how national guidance should relate to a European guidance framework on eIC, it was raised that a European framework could be the guiding star to design national guidance. Therefore, many participants across stakeholder groups considered it important to not exhaustively describe all eIC-related aspects in a European guidance framework in order to enable the EU Member States to incorporate their jurisdictional requirements in national guidance.

“*In national guidance, the level of rolled out technologies should be considered, for example, related to electronic identity systems.*” (2, investigator).

However, many participants across stakeholder groups emphasized that harmonization at European level is the way forward. Therefore, it was argued that the information laid down in national guidance must not conflict with the requirements defined by a European guidance framework. In addition, a regulator and a pharmaceutical industry representative voiced the opinion that it is important to provide insights into the topics for which harmonization cannot be sought.

“*A European guidance framework should contain principles on which the Member States agree. Based on these principles, national initiatives may emerge. However, we need to investigate how we can harmonize as much as possible across Member States.*” (16, regulator).

### Addressing particular aspects in a European guidance framework

#### Definition of electronic informed consent

Generally, all stakeholders agreed with the definition of eIC issued by the EMA as well as the FDA. A few regulators raised the point that, when reference is made to eIC, it may be unclear whether the eIC process occurs at the research site or remotely and whether it concerns a hybrid combination of paper and electronic consent elements. Therefore, it was suggested to further address these distinctions in an eIC definition. In addition, some participants mentioned that the definitions of eIC lack an explicit understanding that eIC applications are designed to support, rather than replace, the interaction between the research team and the research participants.

“*The definitions are essentially very good. In my mind the one thing that both of these definitions [EMA and FDA definition] are missing is that eIC should not replace the physician-patient communication.*” (1, data infrastructure representative).

Moreover, one pharmaceutical industry representative was of the opinion that the definitions stick to a static rather than a longitudinal, interactive eIC process.

“*Based on these definitions, a scanned paper-based informed consent form would also be considered an eIC.”* (17, pharmaceutical industry representative).

#### Legality

Many stakeholders mentioned that the statement of the FDA, referring to the relevant regulatory requirements related to eIC, is the way forward. According to some EC representatives, regulators, and representatives of the pharmaceutical industry, the description of the EMA, stating that “*the sponsor should clarify legality and Good Clinical Practice compliance with each country’s ECs and national regulatory authorities*” is due to the structure and functioning of the EU.

“*I think this is the only way that the EMA can move forward. The EMA statement is due to the structure of the European Union and is a respectful approach with regard to the national regulatory authorities*.” (12, regulator).

Nevertheless, participants pointed out that this description results in a complex and challenging environment to successfully adopt eIC in clinical research. A pharmaceutical industry representative raised that the varying acceptability across EU Member States adds complexity and resource costs. Therefore, participants across stakeholder groups stressed that a European guidance framework should clearly specify the legal acceptance across EU Member States. However, providing up-to-date information was considered challenging by a few participants because the legality of eIC across EU Member States may change over time.

One particular regulator mentioned that when it is legally accepted to use eIC in clinical research, it may be unclear that other issues need to be taken care of, for example, related to identity verification in case of remote consenting. Therefore, it was suggested to list all elements that need to be considered when implementing eIC in a clinical trial.

#### Documents to be submitted to ethics committees

Stakeholders reported differing opinions on the EMA and FDA statements. Most participants across stakeholder groups agreed with the FDA guidance, stating that all information materials the research participants will receive, including multimedia elements, must be submitted to the EC. According to the EMA, “*consideration should be given as to how the system would be presented documentarily to the EC for approval such that it captures the functionality of the systems and the experience of the potential trial participant using it*”. The data infrastructure organization representative and some regulators, EC representatives, and representatives of the pharmaceutical industry raised that this description is too vague and may lead to different interpretations.

“*Most of the time it is better to be clear. A broad description leaves room for interpretation and can be a point of conflict or can result in misunderstandings.*” (12, regulator).

The fact that the EMA refers to the experience of the potential trial participant when using an eIC system was recognized as positive by various EC representatives, patient organization representatives, regulators, and the investigator. According to these stakeholders, it is important to pay attention to the user-friendliness of the system and its functionalities when assessing eIC. A few EC representatives raised that the eIC system can be presented by using screenshots. However, they pointed out that these screenshots, despite the valuable insights they provide, do not allow them to engage with the system in the same way a participant will.

“*You can get a lot of information from the screenshots, except for the interaction*.” (11, EC member).

The data infrastructure organization representative and a few representatives of the pharmaceutical industry argued that storyboards (i.e., visual roadmaps of multimedia elements) could be submitted to ECs. These storyboards could be supplemented with an attestation letter confirming that the content of eIC will be identical to the paper-based consent submitted for EC review. According to these representatives, this approach would support an efficient process since the digitization of the paper-based consent can already take place during EC review. On the other hand, some EC representatives argued that they would like to have access to the eIC application. One particular EC representative added that there was limited experience with assessing eIC, and therefore, it is key to take an in-depth look into the application.

“*I believe that we should receive exactly what the patient receives and we should be able to assess how the patient will navigate through the eIC.*” (8, EC member).

#### Responsibilities of ethics committees

The EMA guideline does not specifically address the responsibilities of ECs in the eIC assessment process whereas the FDA guidance mentions that the EC should look into aspects such as any optional questions to investigate the participants’ understanding of study-related information, the usability of the eIC application to ensure that it is easy to navigate, and how the electronic signature is created. Several participants thought that the EC responsibilities are defined on national level and are therefore not included in the EMA guideline.

“*I think the EMA does not have a description of the responsibilities because it is already defined on national level.*” (6, EC representative).

Nevertheless, the majority of participants argued that, if possible, the responsibilities of an EC should be included in a European guidance framework to avoid inconsistent expectations amongst different ECs. Most of the participants agreed with the responsibilities listed in the FDA guidance. Nevertheless, one pharmaceutical industry representative raised that reviewing optional questions to gauge subject comprehension fits better with the investigator’s responsibility. In addition, a few participants raised that it is important to keep in mind whether ECs will be able to fulfill the responsibilities if these would be listed.

#### Conduct of the electronic informed consent process

Almost all participants across stakeholder groups agreed with the statement of the EMA on where and how the eIC process can take place. According to this statement, it can be “*conducted in person or, it could be done remotely where this can be justified and where allowed nationally and if approved by an EC using electronic methods that allow for two-way communication in real time”.* One pharmaceutical industry representative raised that this description is too vague and that further clarification is needed to know what is exactly allowed. The FDA describes that in-person discussions with the research team can be used to “*ask questions about the study before signing consent as well as at any time during the subject’s involvement in the research*”. In addition, the FDA also refers to electronic means such as video conferencing, electronic messaging, telephone calls, or a live chat to interact with the research team. Stakeholders’ main views on the descriptions of the EMA and the FDA are presented in Table 2. According to many participants, a European guidance framework should emphasize the possibility to interact with the research team physically. However, participants should also be able to discuss study-related information or additional questions only electronically, based on their preferences.

“*I think it depends on the study. Physical contact should always be offered but there must be the chance to contact the investigator electronically.*” (2, investigator).

**Table 2:** Perspectives of stakeholders on the EMA and FDA descriptions

The conduct of the eIC process	
EMA description	FDA description
**Positive elements**: • Emphasis on EC approval in case of a remote interview in which the investigator informs the participants about the pertinent aspects of a trial • Emphasis on electronic methods that allow real-time communication for conducting the interview • Reference to national legislation	**Positive elements**: • Emphasis on the eIC process (i.e., having the possibility to ask questions before and during the conduct of the trial) • Flexibility because the description refers to in-person discussions as well as multiple electronic options to discuss questions with the investigator
**Negative elements**: • No concrete options are provided for how the interview can be organized	**Negative elements**: • Reference is made to the use of electronic methods that do not necessarily allow real-time communication (e.g., electronic messaging)

## Discussion

A scattered regulatory and legal landscape poses challenges for the implementation of eIC in clinical research [9, 13, 23]. The EMA draft ‘Guideline on computerised systems and electronic data in clinical trials’ may play a vital role in advancing the adoption of computerized systems, including eIC, in clinical research [8]. However, further discussion and a more holistic guidance framework, specifically focused on eIC, is needed to advance its successful deployment in clinical research. To this end, semi-structured interviews and focus group discussions were conducted with key stakeholder groups to address identified issues and practical elements in order to support the development of a European guidance framework on eIC. Future activities could be centered around the recommendations outlined in Table 3.

**Table 3:** Multi-stakeholder recommendations for the creation of a European guidance framework on eIC in clinical research

**Overarching recommendations regarding the development of European guidance framework** 1. Involve a multi-stakeholder taskforce to guide the creation of a European guidance framework, with a neutral stakeholder preferably taking the lead
2. Develop a European guidance framework with a high legal value, starting from recommendations or good research practices that may ultimately result in legislation 3. Aim to harmonize as much as possible and to report transparently about the elements for which and the reasons why harmonization cannot be achieved
**Recommendations regarding the content of a European guidance framework** 4. Address specific types of eIC (e.g., a combination of paper and electronic consent elements may be used) 5. Report and update the legal acceptance of eIC across EU Member States
6. Include an overview of the responsibilities of ECs and preferably the documents to be submitted to ECs
7. Emphasize that eIC applications are designed to support the interaction between the research participants and the research team
8. Emphasize the interactive and flexible character of eIC applications

### Towards a unified understanding of electronic informed consent

A few regulators highlighted that it may be unclear what exactly eIC is referring to. eIC applications are often highly configurable. For example, they can be used in a fully remote setting or a combination of eIC elements and obtaining the participants’ wet-ink signature can be employed [24–27]. To avoid misconceptions of what eIC could exactly entail, it is advisable to address these specific types of eIC in a European guidance framework. In addition, as suggested by some stakeholders involved in this study, it should be mentioned that eIC is not meant to replace the personal interaction between the research participants and the research team. Previous research showed that this is one of stakeholders’ main concerns with regard to eIC adoption [9, 10]. This is likely influenced by their limited experience with eIC which, in turns, leads to a lack of awareness of its potential and functioning. Hence, clarification in issued guidance and the broader European guidance framework for eIC is essential.

### The need for a coordinated multi-stakeholder collaboration

All stakeholders in this study highlighted the need for a multi-stakeholder approach when it comes to designing a European guidance framework on eIC, preferably coordinated by the EMA or the CTEG. In 2021, the CTEG, consisting of representatives of national competent authorities and ECs, reported their involvement in setting up a guidance on decentralized clinical trials [28, 29]. As a result, together with the Clinical Trial Coordination Group of the Heads of Medicines Agencies (HMA) and the Good Clinical Practice Inspectors Working Group of the EMA, they issued a Recommendation paper on decentralized elements in clinical trials in December 2022 [30]. In addition, other initiatives are ongoing to facilitate transformation of the design and conduct of clinical trials in view of the emerging technologies. For example, the International Council for Harmonisation of Technical Requirements for Pharmaceuticals for Human Use (ICH) E6(R3) Expert Working Group, a multi-stakeholder collaboration, issued a draft version of updated ICH E6 principles in 2021 [31, 32]. The revision of the E6(R2) Good Clinical Practice guideline aims to, among others, facilitate the use of digital health technologies in clinical trials [32]. The availability of guidance documents, informed by key stakeholders in the field, may lay the foundation to successfully advance the conduct of clinical trials. However, it remains unclear to what extent these guidance documents will cover practical elements regarding eIC.

### Further adoption of electronic informed consent in clinical research

Frameworks play an important role in clinical research and act as safeguards in regulating how stakeholders should undertake their responsibilities [14, 33]. A European guidance framework on eIC may be the way forward to facilitate its implementation. However, even if such a framework would be in force, the adequacy of available infrastructure may affect the successful implementation of eIC in clinical research. A recent survey conducted by TransCelerate, a non-profit organization involving biopharmaceutical research and development organizations, found that one of the biggest challenges with implementing eIC is a heavy resource demand to support its development, approval, and maintenance [23]. Due to the variation across eIC solutions on the market, additional complexity to research operations may be added [34–36]. Using an eIC solution in a clinical study requires training of research staff and the development of standard operating procedures, and thus may be considered burdensome [37]. Another implementation challenge of eIC is related to achieving interoperability with other healthcare applications [38]. To this end, a guidance framework could emphasize the importance of standards, such as Health Level 7 (HL7) Fast Healthcare Interoperability Resources (FHIR), to design eIC solutions that can connect to other systems in order to ease the burden for the site. In the draft guideline issued by the EMA, it is stated that the legality of eIC should be clarified with the national regulatory authorities, which respects the sovereign rights of EU Member States [8]. Nevertheless, when conducting multi-country clinical trials, the country-specific requirements should be understood, for example, related to the use of electronic signatures and the interaction between research participants and the research team [39]. To this end, the abovementioned 2022 EMA/European Commission/HMA joint Recommendation paper on decentralized elements in clinical trials may be an important tool to increase transparency on the legality of eIC [30]. Furthermore, based on consultations with regulatory bodies as well as available literature, an overview of the acceptance of electronic signatures for obtaining informed consent for participation in a study and for data processing could be compiled [13, 30, 40]. However, to facilitate the conduct of these trials, an appropriate way forward would be to harmonize the eIC process as much as possible across EU Member States. Therefore, a fundamental debate is required about what principles should underpin harmonization and how it can be achieved. The topics for which it is not possible to achieve harmonization should be transparently described in a European guidance framework and may be further addressed in national guidance.

### The assessment process by ethics committees

ECs are independent bodies in EU Member States that are empowered to give opinions for the purposes of the CTR [14]. Their main responsibility is to protect the rights, safety, and wellbeing of research participants. Therefore, ECs have a key role in assessing the (electronic) informed consent form [33]. Due to their independent nature, a uniform assessment of eIC is lacking. The CTR, applicable since 21 January 2022, aims to simplify and harmonize the application and evaluation procedures for clinical trials [14, 41]. According to the CTR, the clinical trial application consists of two parts. Part I contains, among other things, the protocol and the investigator’s brochure and will be reviewed by one reporting EU Member State taking into account considerations expressed by the other Member States involved. On the contrary, part II, consisting of other aspects such as the informed consent form and informed consent procedure, will be assessed by each Member state concerned for its own territory [14, 42]. Therefore, structured ethical evaluation of eIC may be a further matter of concern. To this end, a European guidance framework could consist of various elements. First, the draft guideline of the EMA on computerized systems and electronic data in clinical trials can lay down general requirements related to eIC. Second, there is a need for a detailed overview of the legality of eIC across EU Member States. This overview could be provided in view of the ‘Accelerating Clinical Trials in the EU’ (ACT EU) initiative which aims to, among others, support the conduct of multi-country clinical trials [43]. Third, practical elements related to the ethical review process of eIC could be addressed, such as the type of materials an EC should have access to (i.e., printscreens or full access to the eIC application). For example, the European Network of Research Ethics Committees (EUREC) could promote the development of harmonized solutions for reviewing eIC [44].

### Strengths and limitations

To the best of our knowledge, this is the first empirical study aiming to investigate and inform the creation of a European guidance framework related to the implementation of eIC in clinical research. Based on focus group discussions and semi-structured interviews with key stakeholder groups involved in clinical research, we were able to capture a diverse range of opinions. However, it should be noted that the results found in qualitative research are bound to the participant sample, and thus, may not allow for generalization. In addition, it is worth mentioning that we were not able to include stakeholders located in Eastern Europe, despite our sustained efforts to do so.

## Conclusion

Whereas the conduct of clinical trials relies on a solid regulatory framework, the regulatory and legal landscape related to eIC in clinical research is scattered. Therefore, this study brought together key stakeholder groups in clinical research in order to investigate and inform the creation of a European guidance framework on eIC. Generally, the results show that there is a need for a European guidance framework, that both addresses practical elements on eIC and aims to harmonize eIC requirements as much as possible across EU Member States. In addition, stakeholders voiced several suggestions such as involving multi-stakeholder partnerships in the creation of a framework and providing overarching insights into the ethical evaluation process of eIC.

## Electronic supplementary material

Below is the link to the electronic supplementary material.

Supplementary Material 1

Supplementary Material 2

Supplementary Material 3

## Data Availability

The datasets generated and/or analysed during the current study are not publicly available due to confidentiality requirements but are available from the corresponding author on reasonable request.

## List of abbreviations

CTEG: Clinical Trials Expert Group
CTR: Clinical Trials Regulation
EC: ethics committee
EMA: European Medicines Agency
eIC: electronic informed consent
EU: European Union
FDA: US Food and Drug Administration
HMA: Heads of Medicines Agencies
ICH: International Council for Harmonisation of Technical Requirements for Pharmaceuticals for Human Use

## References

[1] U.S. Department of Health & Human Services. National Institutes of Health. NIH’s definition of a clinical trial. 2017. https://grants.nih.gov/policy/clinical-trials/definition.htm. Accessed 26 July 2022.

[2] European Medicines Agency. Clinical trials in human medicines. https://www.ema.europa.eu/en/human-regulatory/research-development/clinical-trials-human-medicines. Accessed 26 July 2022.

[3] Bentley C, Cressman S, van der Hoek K, Arts K, Dancey J, Peacock S (2019). Conducting clinical trials-costs, impacts, and the value of clinical trials networks: a scoping review. Clin Trials.

[4] European Federation of Pharmaceutical Industries and Associations Clinical Research Expert Group. Innovation in clinical trial design: a review of the clinical trial design landscape. 2020. https://www.efpia.eu/media/547507/efpia-position-paper-innovation-in-clinical-trial-design-white-paper.pdf. Accessed 26 July 2022.

[5] Park JJH, Siden E, Zoratti MJ, Dron L, Harari O, Singer J (2019). Systematic review of basket trials, umbrella trials, and platform trials: a landscape analysis of master protocols. Trials.

[6] U.S. Department of Health & Human Services, Food and Drug Administration, Center for Drug Evaluation and Research, Center for Biologics Evaluation and Research, Center for Devices and Radiological Health, Oncology Center of Excellence. Digital health technologies for remote data acquisition in clinical investigations. Guidance for industry, investigators, and other stakeholders. Draft guidance. 2021. https://www.fda.gov/regulatory-information/search-fda-guidance-documents/digital-health-technologies-remote-data-acquisition-clinical-investigations. Accessed 26 July 2022.

[7] de Jong AJ, van Rijssel TI, Zuidgeest MGP, van Thiel GJMW, Askin S, Fons-Martínez J (2022). Opportunities and Challenges for decentralized clinical trials: european regulators’ perspective. Clin Pharmacol Ther.

[8] European Medicines Agency. Good Clinical Practice Inspectors Working Group (GCP IWG). Guideline on computerised systems and electronic data in clinical trials - Draft. 2021. https://www.ema.europa.eu/en/documents/regulatory-procedural-guideline/draft-guideline-computerised-systems-electronic-data-clinical-trials_en.pdf.

[9] De Sutter E, Borry P, Geerts D, Huys I (2021). Personalized and long-term electronic informed consent in clinical research: stakeholder views. BMC Med Ethics.

[10] De Sutter E, Zaçe D, Boccia S, Di Pietro ML, Geerts D, Borry P (2020). Implementation of electronic informed consent in Biomedical Research and Stakeholders’ Perspectives: systematic review. J Med Internet Res.

[11] De Sutter E, Geerts D, Borry P, Coteur K, Bamps D, Marynissen H (2022). Co-creation with research participants to inform the design of electronic informed consent. Digit HEALTH.

[12] TransCelerate Biopharma INC, eConsent. Implementation guidance. 2017. https://www.transceleratebiopharmainc.com/initiatives/econsent/.

[13] De Sutter E, Meszaros J, Borry P, Huys I. Digitizing the informed consent process: a review of the Regulatory Landscape in the European Union. Front Med. 2022;9. 10.3389/fmed.2022.906448. 10.3389/fmed.2022.906448 PMC9174519 35692551

[14] Regulation (EU). No 536/2014 of the European Parliament and of the Council of 16 April 2014 on clinical trials on medicinal products for human use, and repealing Directive 2001/20/EC (2014).

[15] Regulation (EU). No 910/2014 of the European Parliament and of the Council of 23 July 2014 on electronic identification and trust services for electronic transactions in the internal market and repealing Directive 1999/93/EC. https://ec.europa.eu/futurium/en/system/files/ged/eidas_regulation.pdf.

[16] U.S. Food and Drug Administration (FDA). Use of electronic informed consent: questions and answers. Guidance for institutional review boards, investigators and sponsors. 2016. https://www.fda.gov/regulatory-information/search-fda-guidance-documents/use-electronic-informed-consent-clinical-investigations-questions-and-answers.

[17] European Medicines Agency. Guidance on the management of clinical trials during the COVID-19 (Coronavirus) pandemic. 2021. https://ec.europa.eu/health/sites/health/files/files/eudralex/vol-10/guidanceclinicaltrials_covid19_en.pdf. Accessed 26 July 2022.

[18] Tong A, Sainsbury P, Craig J (2007). Consolidated criteria for reporting qualitative research (COREQ): a 32-item checklist for interviews and focus groups. Int J Qual Health Care.

[19] Tausch AP, Menold N (2016). Methodological aspects of Focus Groups in Health Research:results of qualitative interviews with Focus Group moderators. Global Qualitative Nursing Research.

[20] Leung FH, Savithiri R (2009). Spotlight on focus groups. Can Fam Physician.

[21] Glaser BG, Strauss AL. The Discovery of grounded theory: strategies for qualitative research. Aldine; 1967.

[22] Gale NK, Heath G, Cameron E, Rashid S, Redwood S (2013). Using the framework method for the analysis of qualitative data in multi-disciplinary health research. BMC Med Res Methodol.

[23] TransCelerate Biopharma Inc.: TransCelerate’s Modernizing Clinical Trial Conduct (MCTC) Initiative. Modern Solution Adoption. Maturity Survey Results. 2021. https://www.transceleratebiopharmainc.com/wp-content/uploads/2021/12/MCTC-Maturity-Survey-Detailed-Report-2021.pdf. Accessed 22 June 2022.

[24] Almeida-Magana R, Maroof H, Grierson J, Clow R, Dinneen E, Al-Hammouri T (2022). E-Consent—a guide to maintain recruitment in clinical trials during the COVID-19 pandemic. Trials.

[25] SignantHealth. SmartSignals eConsent. https://www.signanthealth.com/solutions/patient-solutions/econsent/. Accessed 20 April 2022.

[26] DrugDev. eConsent. https://www.drugdev.com/solutions/drugdev-econsent/. Accessed 20 April 2022.

[27] Medidata. eConsent. https://www.medidata.com/en/clinical-trial-services/patient-centric-clinical-trials/econsent. Accessed 20 April 2022.

[28] European Commission. Directorate-General for Health and Food Safety, Meeting of the Expert Group on Clinical Trials. Draft meeting minutes 14 December 2021. 2021. https://ec.europa.eu/transparency/expert-groups-register/screen/expert-groups/consult?do=groupDetail.groupDetail&groupID=1464&NewSearch=1&NewSearch=1. Accessed 29 July 2022.

[29] European Commission. Register of Commission Expert Groups and Other Similar Entities. Expert group on clinical trials (E01464). https://ec.europa.eu/transparency/expert-groups-register/screen/expert-groups/consult?do=groupDetail.groupDetail&groupID=1464&NewSearch=1&NewSearch=1. Accessed 12 August 2022.

[30] Heads of Medicines Agencies, European Commission, European Medicines Agency. Recommendation paper on decentralised elements in clinical trials. 2022. https://health.ec.europa.eu/system/files/2022-12/mp_decentralised-elements_clinical-trials_rec_en.pdf. Accessed 31 January 2023.

[31] The International Council for Harmonisation of Technical Requirements for Pharmaceuticals for Human Use. ICH E6 Guideline for Good Clinical practice (GCP) - Update on progress. Public web conference report. 2021. https://database.ich.org/sites/default/files/ICH_E6R3_WebConference_Report_Final_2021_1011.pdf. Accessed 12 August 2022.

[32] The International Council for Harmonisation of Technical Requirements for Pharmaceuticals for Human Use. ICH-E6 Good Clinical Practice (GCP). Explanatory Note. 2021. https://database.ich.org/sites/default/files/ICH_E6-R3_GCP-Principles_Draft_2021_0419.pdf.

[33] The International Council for Harmonisation of Technical Requirements for Pharmaceuticals for Human Use. Integrated addendum to ICH E6(R1): Guideline for Good Clinical Practice E6(R2). 2016. https://database.ich.org/sites/default/files/E6_R2_Addendum.pdf.

[34] Complete Consent IQVIAIQVIA. 2022. https://www.iqvia.com/-/media/iqvia/pdfs/library/brochures/iqvia-complete-consent.pdf?_=1667329445992.

[35] ICON plc. eConsent: enhanced informed consent. https://www.iconplc.com/innovation/firecrest/automate/econsent/.

[36] SignantHealth. Signant SmartSignals eConsent. Flexible solutions to facilitate comprehension and compliance. https://www.signanthealth.com/wp-content/uploads/2021/06/Tiered-eConsent-Clinical-Trial-Solutions.pdf.

[37] Bromberg JR, Nimaja E, Kiragu AW, Lawson KA, Lee L, Nasr IW (2022). Developing and implementing electronic consent procedures in response to Covid-19 restrictions. Ethics Hum Res.

[38] Verreydt S, Yskout K, Joosen W (2021). Security and privacy requirements for electronic consent: a systematic literature review. ACM Trans Comput Healthcare.

[39] Thiers FA, Sinskey AJ, Berndt ER (2008). Trends in the globalization of clinical trials. Nat Rev Drug Discovery.

[40] Dierks C, Kircher P, Husemann C, Kleinschmidt J, Haase M. Data privacy in European Medical Research: a contemporary legal opinion. Medizinisch Wissenschaftliche Verlagsgesellschaft; 2021.

[41] European Medicines Agency. Clinical Trials Regulation. https://www.ema.europa.eu/en/human-regulatory/research-development/clinical-trials/clinical-trial-regulation. Accessed 1 August 2022.

[42] Federal Agency for Medicines and Health Products. Guidance document for clinical trial sponsors. Voluntary Joint pilot project between FAMHP, the College, accredited Ethics Committees and sponsors for processing of applications for the authorisation of clinical trials and substantial modifications on medicinal products for human use in accordance with the spirit of the Regulation (EU) No 536/2014 and of the law on Clinical Trial Regulation (CTR). Version 9.1. 2021. https://www.fagg.be/sites/default/files/new%20version%20procedure.pdf. Accessed 1 August 2022.

[43] Heads of Medicines Agencies, European Commission, European Medicines Agency. Accelerating Clinical Trials in the EU (ACT EU): Delivering an EU clinical trials transformation initiative. 2022. https://www.ema.europa.eu/en/documents/regulatory-procedural-guideline/accelerating-clinical-trials-eu-act-eu-delivering-eu-clinical-trials-transformation-initiative_en.pdf. Accessed 6 October 2022.

[44] European Network of Research Ethics Committees - EUREC. http://www.eurecnet.org/index.html. Accessed 6 October 2022.

