# Supplementary material for: Informing a European guidance framework on electronic informed consent in clinical research: a qualitative study
Source: BMC Health Serv Res. 2023 Feb 21;23:181. doi: 10.1186/s12913-023-09173-5 (PMC9942635; doi:10.1186/s12913-023-09173-5)
Supplement: Supplementary file 1 — Supplementary Material 1 [file 12913_2023_9173_MOESM1_ESM.docx]

**Informing a European guidance framework on electronic informed consent in clinical research: a qualitative study**

Evelien De Sutter*^1^, Pascal Borry^2^, Isabelle Huys^1+^, Liese Barbier^1+^

^1^Clinical Pharmacology and Pharmacotherapy, Department of Pharmaceutical and Pharmacological Sciences, KU Leuven, Leuven, Belgium

^2^Centre for Biomedical Ethics and Law, Department of Public Health and Primary Care, KU Leuven, Leuven, Belgium

**Corresponding author*

*^+^These authors share last authorship*

## Additional file 1: COREQ checklist

| **Item and guide questions** | **Response** | **Location in manuscript (Section)** |
| --- | --- | --- |
| **Domain 1: Research team and reﬂexivity** | | |
| **Personal Characteristics** | | |
| 1. Interviewer/facilitator:  Which author/s conducted the interview or focus group? | Moderators: EDS or JD.  Observers (in the focus group discussions only): LB and EDS or JD. | Methods |
| 2. Credentials:  What were the researcher’s credentials? E.g., PhD, MD | EDS: PharmD  JD: Master’s student in biomedical sciences  LB: PharmD, PhD | - |
| 3. Occupation:  What was their occupation at the time of the study? | At the time of the study, EDS working towards her PhD and JD towards his Master dissertation at KU Leuven. LB held a postdoctoral position. | - |
| 4. Gender:  Was the researcher male or female? | Female (EDS and LB) and male (JD). | - |
| 5. Experience and training:  What experience or training did the researcher have? | EDS and LB had previous experience in conducting interviews, workshops, and focus group discussions. | - |
| **Relationship with participants** | | |
| 6. Relationship established:  Was a relationship established prior to study commencement? | Prior to the interview or focus group discussion, participants were briefed on the study and were provided with the informed consent. | Methods |
| 7. Participant knowledge of the interviewer:  What did the participants know about the researcher? | The participants were informed that EDS, LB, and JD were researching electronic informed consent at KU Leuven. | Methods |
| 8. Interviewer characteristics:  What characteristics were reported about the interviewer/facilitator? | The background of the moderator and the observer(s), if applicable. | Methods |
| **Domain 2: Study design** | | |
| **Theoretical framework** | | |
| 9. Methodological orientation and theory:  What methodological orientation was stated to underpin the study? | Semi-structured interviews and focus group discussions that were analyzed using a framework method. | Methods |
| **Participant selection** | | |
| 10. Sampling:  How were participants selected? | Through purposive and snowballing sampling. | Methods |
| 11. Method of approach:  How were participants approached? | Via mail. | Methods |
| 12. Sample size:  How many participants were in the study? | In total, 20 participants had taken part in this study. | Results |
| 13. Non-participation:  How many people refused to participate or dropped out? Reasons? | 74% of invited individuals refused to participate. Reasons included unavailability and non-response to the invitation. | - |
| **Setting** | | |
| 14. Setting of data collection:  Where was the data collected? | Interviews and focus group discussions took place remotely. | Methods |
| 15. Presence of non-participants:  Was anyone else present besides the participants and researchers? | No | Methods |
| 16. Description of sample:  What are the important characteristics of the sample? | Participants belong to one of the six stakeholder groups. They were active across European Union Member States or at a pan-European or global level. | Results |
| **Data collection** | | |
| 17. Interview guide:  Were questions, prompts, guides provided by the authors? Was it pilot tested? | The topic guide was provided to the participants. The questions were not pilot tested. | Methods |
| 18. Repeat interviews:  Were repeat interviews carried out? If yes, how many? | No | - |
| 19. Audio/visual recording:  Did the research use audio or visual recording to collect the data? | Digital audio recordings of all interviews and focus group discussions were made. | Methods |
| 20. Field notes:  Were ﬁeld notes made during and/or after the interview or focus group? | Yes | Methods |
| 21. Duration:  What was the duration of the interviews or focus groups? | Interviews: 30 - 50 min long  Focus group discussions: 1 - 2 hours long | Methods |
| 22. Data saturation:  Was data saturation discussed? | No | - |
| 23. Transcripts returned:  Were transcripts returned to participants for comment and/or correction? | No | - |
| **Domain 3: Analysis and ﬁndings** | | |
| **Data analysis** | | |
| 24. Number of data coders:  How many data coders coded the data? | The first transcript was coded by one researcher (JD). However, the defined codes were discussed and approved by two other researchers (EDS and LB). The other transcripts were coded by one researcher (EDS). | Methods |
| 25. Description of the coding tree:  Did authors provide a description of the coding tree? | Coding was described in the methods section. Moreover, the coding tree is reported in the supplementary material accompanying the manuscript. | Methods – Supplementary material |
| 26. Derivation of themes:  Were themes identified in advance or derived from the data? | Transcripts were coded by using a combination of an inductive and deductive approach. | Methods |
| 27. Software:  What software, if applicable, was used to manage the data? | NVivo and Microsoft Excel. | Methods |
| 28. Participant checking:  Did participants provide feedback on the findings? | No | - |
| **Reporting** | | |
| 29. Quotations presented:  Where participant quotations presented to illustrate the themes/findings? Was each quotation identified? | Yes | Results |
| 30. Data and ﬁndings consistent:  Was there consistency between the data presented and the findings? | Yes | Results |
| 31. Clarity of major themes:  Were major teams clearly presented in the findings? | Results are presented per major theme. | Results |
| 32. Clarity of minor themes:  Is there a description of diverse cases or discussion of minor themes? | Minor themes are discussed in the manuscript, for example by using quotations. | Results |
