# Supplementary material for: Informing a European guidance framework on electronic informed consent in clinical research: a qualitative study
Source: BMC Health Serv Res. 2023 Feb 21;23:181. doi: 10.1186/s12913-023-09173-5 (PMC9942635; doi:10.1186/s12913-023-09173-5)
Supplement: Supplementary file 2 — Supplementary Material 2 [file 12913_2023_9173_MOESM2_ESM.docx]

**Informing a European guidance framework on electronic informed consent in clinical research: a qualitative study**

Evelien De Sutter*^1^, Pascal Borry^2^, Isabelle Huys^1+^, Liese Barbier^1+^

^1^Clinical Pharmacology and Pharmacotherapy, Department of Pharmaceutical and Pharmacological Sciences, KU Leuven, Leuven, Belgium

^2^Centre for Biomedical Ethics and Law, Department of Public Health and Primary Care, KU Leuven, Leuven, Belgium

**Corresponding author*

*^+^These authors share last authorship*

## Additional file 2: Topic guide focus group discussions and semi-structured interviews

**I. General introduction**

- Welcoming the participant(s)
- Introducing the moderator(s), observers (if applicable) and the research study
- Explaining the general rules of the focus group discussion or semi-structured interview

**II. Introduction to the topic of electronic informed consent**

The US Food and Drug Administration (FDA) as well as the European Medicines Agency (EMA) issued definitions of electronic informed consent.

- **Definition of the FDA**:

*“Electronic informed consent refers to the use of electronic systems and processes that may employ multiple electronic media, including text, graphics, audio, video, podcasts, passive and interactive Web sites, biological recognition devices, and card readers, to convey information related to the study and to obtain and document informed consent.”*

- **Definition of the EMA**:

“*An electronic informed consent refers to the use of any digital media (e.g. text, graphics, audio, video, podcasts or websites) to firstly convey information related to the clinical trial to the trial participant and secondly document informed consent via an electronic device (e.g., mobile phones, tablets or computers)*.”

Both definitions are more or less similar. What do you think of these definitions?

**III. Type of European guidance framework**

- When you first heard of a European guidance framework related to the implementation or use of electronic informed consent in clinical research, what was the first thing that came to your mind? What kind of European guidance framework would be needed?
- What type of value should this guidance framework ideally have?
- Who (which stakeholder(s)) should initiate the creation of this European guidance framework?
- The Good Clinical Practice Inspectors Working Group of the EMA issued a draft guideline on computerized systems and electronic data in clinical trials. What do you think of this guidance? What do you think the impact of this guidance could be on adoption of electronic informed consent in clinical research?
- How should national guidance relate to European guidance?

**IV. A European guidance framework: detailed discussion of topics**

**A) Harmonization of electronic informed consent**

The first topic that we would like to discuss is related to the legality of electronic informed consent. Participants in one of our research studies supported harmonization of electronic informed consent across European Union Member States and beyond. However, they indicated that establishing a harmonized approach would be challenging.

1) Legality

- **FDA guidance: “***This guidance clarifies that when implementing an eIC, a variety of approaches may be used to fulfill HHS and FDA regulatory requirements for informed consent and IRB review (45 CFR part 46 and 21 CFR parts 50 and 56) and FDA regulations for electronic records and electronic signatures (21 CFR part 11).*”
- **EMA guidance: “***Before implementation of an electronic consent procedure is considered, the sponsor should clarify legality and GCP compliance with each country’s ethics committees and national regulatory authorities.*”

The FDA guidance refers to the relevant regulatory requirements to support compliant use of electronic informed consent. The EMA guidance indicates that legality must be clarified with national regulatory authorities. What is your opinion on this difference? What could be measures to facilitate the clarification of legality across European Union Member States?

**B) Ethical review of electronic informed consent**

2) Which materials need to be submitted to the relevant ethics committee?

- **FDA guidance**: “*The investigator should submit to the IRB copies of all forms (electronic and paper forms) and informational materials, including any videos and Web-based presentations, which the subject will receive and view during the eIC process*.”
- **EMA guidance**: “*Consideration should be given as to how the system would be presented documentarily to the EC for approval such that it captures the functionality of the systems and the experience of the potential trial participant using it.*”

The FDA guidance describes in a detailed way which materials need to be submitted to the ethics committee, whereas the guidance of EMA leaves some room for interpretation. Which description would you prefer? What would you have to change for a European framework?

3) What are the responsibilities of the relevant ethics committee in the evaluation process of electronic informed consent?

- **FDA guidance**: “*A critical part of this responsibility is for the IRB to ensure there is an adequate informed consent process that protects the rights and welfare of subjects participating in HHS-regulated research and FDA-regulated clinical investigations. Therefore, the IRB must review and approve the eIC and any amendments to the eIC that the subject will receive and view. The IRBs should also review any optional questions or methods used to gauge subject comprehension of key study elements. The IRB should also review the usability of the eIC materials to ensure that they are easy to navigate. If the program uses hyperlinks to convey study-related information, IRBs should review the contents to which subjects are referred in order to determine if the study-related information that has been supplied is accurate and appropriate. Because Web sites are often modified over time, IRBs must maintain the version of the Web site information that contains the study-related information that the IRB reviews and approves, either electronically or as a hard copy. IRBs, investigators, and sponsors should consider such issues as how the electronic signature is created and whether the informed consent or permission document can be produced in hard copy for review by the subject upon request. IRBs, investigators, and sponsors may rely on a statement from the vendor of the electronic system used for obtaining the electronic signature that describes how the signature is created and that the system meets the relevant requirements contained in 21 CFR part 11.”*
- **EMA guidance:** /

Only the guidance of the FDA provides information on the responsibilities of the ethics committee when evaluating electronic informed consent. What is your opinion on incorporating the responsibilities of the ethics committee in a European guidance framework? Which responsibilities do you think that ethics committees should have?

**C) Conduct of the electronic informed consent process**

4) Where can the electronic informed consent process take place?

- **FDA guidance: “***The investigator should have methods in place to ensure that the eIC process allows subjects the opportunity to consider whether or not to participate and to ask questions about the study before signing consent as well as at any time during the subject’s involvement in the research.* *This may be accomplished by in-person discussions with study personnel or through a combination of electronic messaging, telephone calls, video conferencing, or a live chat with a remotely located investigator or study personnel.”*
- **EMA guidance:** “*The interview should be conducted in person or, it could be done remotely where this can be justified and where allowed nationally and if approved by an ethics committee using electronic methods that allow for two-way communication in real time*.”

What do you think of the proposed options? What are the pros en cons?

**V. Other considerations and closing**

- We discussed several topics. Do you have any further comments or issues which you think may be relevant to a European guidance framework?
- Do you have any questions?
