# Supplementary material for: Informing a European guidance framework on electronic informed consent in clinical research: a qualitative study
Source: BMC Health Serv Res. 2023 Feb 21;23:181. doi: 10.1186/s12913-023-09173-5 (PMC9942635; doi:10.1186/s12913-023-09173-5)
Supplement: Supplementary file 3 — Supplementary Material 3 [file 12913_2023_9173_MOESM3_ESM.docx]

**Informing a European guidance framework on electronic informed consent in clinical research: a qualitative study**

Evelien De Sutter*^1^, Pascal Borry^2^, Isabelle Huys^1+^, Liese Barbier^1+^

^1^Clinical Pharmacology and Pharmacotherapy, Department of Pharmaceutical and Pharmacological Sciences, KU Leuven, Leuven, Belgium

^2^Centre for Biomedical Ethics and Law, Department of Public Health and Primary Care, KU Leuven, Leuven, Belgium

**Corresponding author*

*^+^These authors share last authorship*

## Additional file 3: Coding tree

| **Code** | **Sub-code level 1** | **Sub-code level 2** |
| --- | --- | --- |
| Definition |  |  |
|  | Suggestions/wishes |  |
| Creation of a guidance framework | First thoughts |  |
|  | Why needed |  |
|  | Type and value |  |
|  | Stakeholders who should be involved in the creation |  |
|  | National framework vs European guidance framework |  |
|  | Impact of EMA guidance |  |
|  | Place of EMA draft guidance in a European guidance framework |  |
| Discussion topics | Legality |  |
|  |  | EU structure |
|  |  | Initiatives to clarify legality |
|  |  | Other elements to be considered |
|  | Ethical review | Ethics committee responsibilities |
|  |  | Documents to be submitted |
|  | Conduct of the electronic informed consent process |  |
|  |  | Positive elements |
|  |  | Negative elements |
